# Nomograms based on SUVmax of ^18^F-FDG PET/CT and clinical parameters for predicting progression-free and overall survival in patients with newly diagnosed extranodal natural killer/T-cell lymphoma

**DOI:** 10.1186/s40644-020-00379-y

**Published:** 2021-01-08

**Authors:** Hongyan Li, Guozhu Shao, Yajing Zhang, Xiaomin Chen, Chengcheng Du, Kun Wang, Zairong Gao

**Affiliations:** 1 Department of Nuclear Medicine, Union Hospital, Tongji Medical College, Huazhong University of Science and Technology, No. 1277 Jiefang Ave, Wuhan, 430022 China; 2 Hubei Province Key Laboratory of Molecular Imaging, Wuhan, 430022 China; 3 Department of Radiology, Union Hospital, Tongji Medical College, Huazhong University of Science and Technology, No. 1277 Jiefang Ave, Wuhan, 430022 China

**Keywords:** ^18^F-FDG PET/CT, SUVmax, Extranodal natural killer/T-cell lymphoma, Nomogram, Prognosis

## Abstract

**Background:**

The prognostic value of ^18^F-FDG PET/CT in extranodal natural killer/T-cell lymphoma (ENKTL) is not well established. We aimed to develop nomograms for individualized estimates of progression-free survival (PFS) and overall survival (OS) in patients with ENKTL using ^18^F-FDG PET/CT parameters and clinical parameters.

**Methods:**

A total of 171 patients with newly diagnosed ENKTL undergoing ^18^F-FDG PET/CT scanning were retrospectively analyzed. Nomograms were constructed according to multivariate Cox proportional hazards regression. The predictive and discriminatory capacities of the nomograms were then measured using the concordance index (C-index), calibration plots, and Kaplan-Meier curves. The C-index, the area under receiver operating characteristic (ROC) curve (AUC), and decision curve analysis (DCA) were used to contrast the predictive and discriminatory capacities of the nomograms against with the International Prognostic Index (IPI) and Korean Prognostic Index (KPI).

**Results:**

Multivariate analysis demonstrated that pretreatment SUVmax≥9.5, disease stage II and III-IV, elevated lactate dehydrogenase (LDH), and elevated β2-microglobulin (β2-MG) had the strongest association with unfavorable PFS and OS. In addition, hemoglobin (Hb) < 120 g/L had a tendency to be associated with PFS. Both nomogram models incorporated SUVmax, Ann Arbor stage, LDH, and β2-MG. The PFS nomogram also included Hb. The nomograms showed good prediction accuracies, with the C-indexes for PFS and OS were 0.729 and 0.736, respectively. The calibration plots for 3-year and 5-year PFS/OS reported good consistency between predicted and observed probabilities for survival time. The PFS and OS were significantly different according to tertiles of nomogram scores (*p* < 0.001). The C-index and AUCs of the nomograms were higher than that of IPI and KPI. Moreover, DCA showed that the predictive accuracy of the nomograms for PFS and OS were both higher than that of IPI and KPI.

**Conclusions:**

This study established nomograms that incorporate pretreatment SUVmax and clinical parameters, which could be effective tools for individualized prognostication of both PFS and OS in patients with newly diagnosed ENKTL.

## Background

Extranodal natural killer/T-cell lymphoma (ENKTL) is recognized as a distinct pathological entity classified by the World Health Organization (WHO) classification of lymphoid tumors [1]. It is a rare subtype of non-Hodgkin lymphoma in Europe and North America; however, it is much more common in Asia and South America [2]. Especially in China, ENKTL is the most common peripheral T-cell lymphoma, accounting for 11–13% of all lymphomas [3, 4]. The cumulative probability of 5-year survival rates range from 30 to 86%, and most studies revealed rates of less than 50% [5]. To date, the prognosis and optimal therapy of ENKTL have not been inadequately defined yet. Therefore, an optimal prognostic model is urgently needed for the early identification of patients at high risk of progression or recurrence and for the improvement of clinical outcomes in ENKTL patients. Currently, the International Prognostic Index (IPI) [6] and Korean Prognostic Index (KPI) [7] were the most common prognostic models. However, the predictive accuracy of those prognostic scoring systems has been reported to be limited for ENKTL [8, 9].

^18^F-fluorodeoxyglucose positron emission tomography/computed tomography (^18^F-FDG PET/CT) plays important roles in staging, assessing treatment response, and prognostication in malignant lymphoma, especially in Hodgkin lymphoma, diffuse large B-cell lymphoma and follicular lymphoma [10–12]. Published experience and recommendations on the role of ^18^F-FDG PET/CT in ENKTL are limited due to the rarity and geographical differences of the disease. There is emerging evidence in a few reports that semiquantitative parameters before treatment, including maximum standardized uptake value (SUVmax), metabolic tumor volume (MTV), and total lesion glycolysis (TLG) may be useful prognostic indicators in patients with ENKTL [13, 14]. However, nomograms consisting of tumor metabolism and clinical parameters for predicting survival estimates for ENKTL patients have not yet been developed. Nomograms are advanced prediction models that can provide a numerical probability of the survival outcome for an individual patient.

In this retrospective study, we aimed to develop prognostic models including the semiquantitative parameter SUVmax and clinical parameters for patients with stage I-IV ENKTL.

## Methods

### Patients

From February 2013 to December 2018, 171 consecutive patients with newly diagnosed ENKTL according to the WHO classification criteria were recruited in this retrospective study. Patients with central nervous system involvement were excluded from the study. Patients who had concomitant infection, had other malignant tumors or had an elevated fasting blood glucose level greater than 200 mg/dL were also excluded from this study.

All included patients underwent pretreatment ^18^F-FDG PET/CT examination. Among them, 63 patients underwent interim PET/CT scans (after 2 to 4 cycles of chemotherapy and/or radiotherapy). Thirty patients underwent end-of-treatment PET/CT scans (after first-line therapy). Interim and end-of-treatment PET/CT scans were carried out 3 weeks after the initial treatment and after the completion of the first-line therapy, respectively.

### PET/CT scan

All patients underwent whole-body (from the skull base to the upper third of the thighs) ^18^F-FDG PET/CT scans with a dedicated PET/CT system (Discovery VCT; GE Healthcare, Milwaukee, WI). After at least 6 h of fasting with blood glucose level lower than 200 mg/dL, an intravenous injection of 3.7–4.4 MBq/kg ^18^F-FDG was administered. Approximately 60 min after the injection of ^18^F-FDG, whole-body CT and PET scans were acquired. Images were corrected for attenuation with low-dose CT data, and corrected PET images were reconstructed using an ordered-subset expectation maximization iterative reconstruction algorithm. The acquired images from CT and PET were reviewed on a dedicated workstation (Xeleris Workstation, GE Healthcare).

### PET/CT image analysis

PET/CT images were retrospectively reviewed by two experienced nuclear medicine physicians, who had information on the initial clinical data but were blinded to the reference standard outcome. The pretreatment images were evaluated by SUVmax, which was recorded in regions of interest drawn over all lesions. We selected the highest SUVmax of images identified for each patient. The interim and end-of-treatment PET/CT data were evaluated based on the Deauville score (DS): 1, no uptake above background; 2, uptake ≤ mediastinum; 3, uptake > mediastinum but ≤ liver; 4, uptake moderately stronger than liver; 5, uptake markedly higher than liver and/or the presence of new lesions [15].

### Statistical analysis

Progression-free survival (PFS) was measured as the period from diagnosis to disease progression, recurrence, death or the last follow-up. Overall survival (OS) was defined from the date of diagnosis to death or the last follow-up. The optimal cut-off values of SUVmax for survival prediction were determined by the receiver operating characteristic (ROC) curve and the area under curve (AUC). Survival curves were analyzed using the Kaplan-Meier method and compared with the log-rank test according to the prognostic factors. The hazard ratio (HR) and 95% confidence intervals (95% CIs) were calculated by the Cox proportional hazards model. Multivariate Cox proportional hazards regression was performed to identify independent predictors for survival outcomes. Backward stepwise selection with the Akaike information criterion (AIC) was used to identify variables for multivariable Cox proportional hazards regression. Selected variables were included in the nomograms for predicting 3-year and 5-year PFS and OS rates of patients with ENKTL.

Discrimination was evaluated by the concordance index (C-index). The model was validated internally, using 300 bootstrap samples. Calibration was evaluated by a calibration plot, which compares the relationship between the predicted and observed probabilities for survival time. The total points for each patient were calculated using the proposed nomograms, and all patients were categorized into three subgroups according to the tertile of their total risk score. Kaplan-Meier survival curves and the log-rank test were used to compare whether the survival distributions differed among the three subgroups. The C-index, ROC curve analysis, and decision curve analysis (DCA) were used to contrast the predictive and discriminatory capacity of the nomograms against with IPI and KPI. Statistical analysis was performed using R version 3.6.1 (http://www.R-project.org). A *p* value less than 0.05 was considered statistically significant.

## Results

### Patient characteristics

The baseline clinical characteristics of the 171 patients enrolled in this study are listed in Table 1. Of these 171 patients, 61 (35.7%) patients had Ann Arbor stage I, 48 (28.1%) had Ann Arbor stage II, 22 (12.8%) had Ann Arbor stage III, and 40 (23.4%) had Ann Arbor stage IV. Most patients (90.1%) had primary disease primarily located in the upper aerodigestive tract (UADT). A total of 49.1% of patients had B symptoms at diagnosis. Elevated lactate dehydrogenase (LDH) and elevated β2-microglobulin (β2-MG) were observed in 38.6 and 34.5% of patients, respectively. Thirty-nine patients (22.8%) had a pretreatment hemoglobin (Hb) level < 120 g/L. The majority (68.4%) of patients were scored as low risk using the IPI, and 63.2% of patients were in groups 1–2 according to the KPI.

**Table 1:** Baseline clinical characteristics of patients

Characteristic	No. patients	Proportion (%)
Sex		
Male	116	67.8
Female	55	32.2
Age		
≤ 60 y	149	87.1
> 60 y	22	12.9
Ann Arbor Stage		
I	61	35.7
II	48	28.1
III	22	12.8
IV	40	23.4
Primary site		
UADT	154	90.1
Extra-UADT	17	9.9
B symptoms		
No	87	50.9
Yes	84	49.1
LDH		
Normal	105	61.4
Elevated	66	38.6
β2-MG		
Normal	112	65.5
Elevated	59	34.5
Hb		
≥ 120 g/L	132	77.2
< 120 g/L	39	22.8
IPI		
Low (0–1)	117	68.4
Intermediate low (2)	33	19.3
Intermediate high (3)	12	7.0
High (≥4)	9	5.3
KPI		
Group 1 (0)	49	28.7
Group 2 (1)	59	34.5
Group 3 (2)	30	17.5
Group 4 (≥3)	33	19.3

*Abbreviations*: *UADT* upper aerodigestive tract, *LDH* lactate dehydrogenase, *β2-MG* β2-microglobulin, *Hb* hemoglobin, *IPI* Prognostic Index, *KPI* Korean Prognostic Index

The median PFS and OS were 34 months and 59 months, respectively, at a median follow-up of 46-months. The 5-year PFS and OS rates for all patients were 33.3 and 45.0%, respectively. Of the 171 patients, 28 patients progressed/relapsed, and 62 patients died of disease progression or recurrence.

### Prognostic factors of PFS and OS

**Fig. 1:**
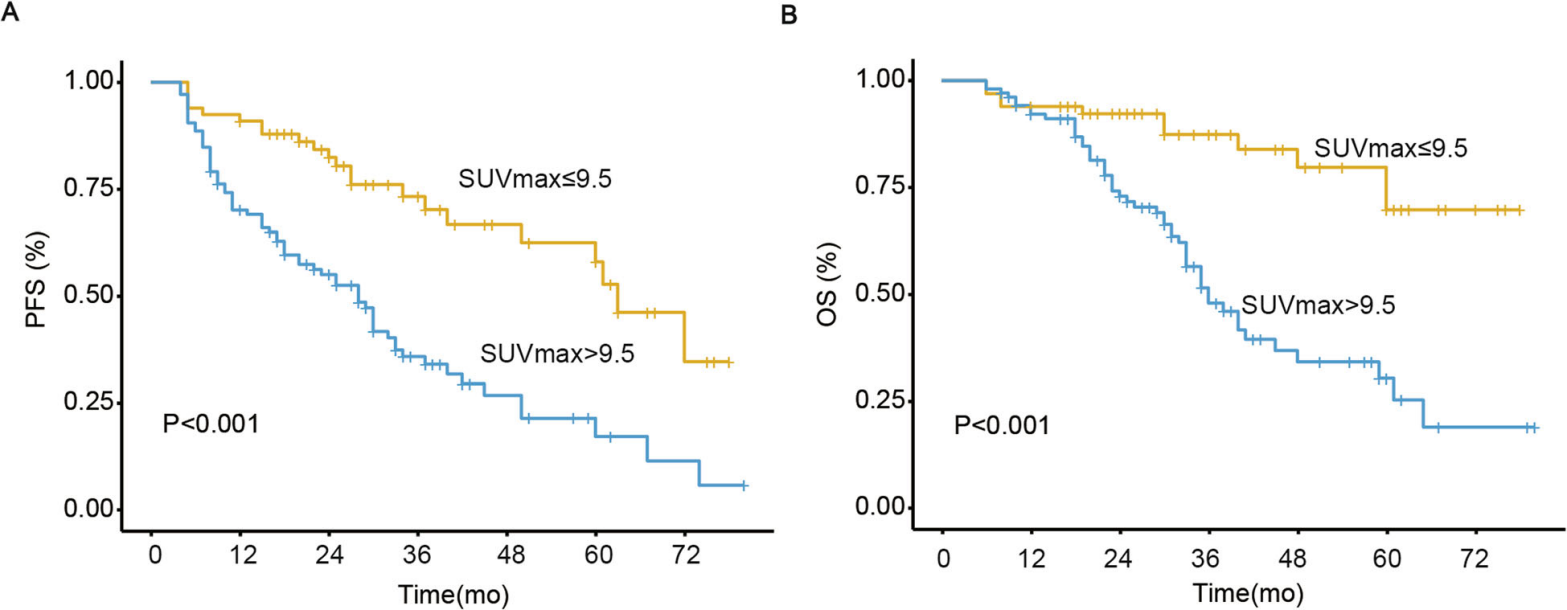
Comparison of Kaplan–Meier survival curves; **a** progression-free survival (PFS) and **b** overall survival (OS) according to pretreatment SUVmax (≤9.5 vs. > 9.5)

In this set of patients, the median SUVmax was 11.4 (range, 1.5–41.4). The optimal SUVmax cut-off value was 9.5 determined by ROC analysis (AUC = 0.698). Using this cut-off value, all patients were divided into high SUVmax (SUVmax ≥9.5, *n* = 105) and low SUVmax (SUVmax < 9.5, *n* = 66) groups. A total of 68 (64.8%) patients in the high SUVmax group experienced treatment failure (progression, recurrence or death), whereas 22 (33.3%) patients in the low SUVmax group experienced treatment failure. The Kaplan-Meier survival curves demonstrated that patients in the high SUVmax group had significantly inferior PFS and OS rates (*p* < 0.001, Fig. 1). The mean PFS and OS in the high SUVmax group were 32.2 ± 2.8 and 43.2 ± 3.0 months, respectively, and those in the low SUVmax group were 54.9 ± 3.7 and 66.0 ± 3.2 months.

In all, 63 patients underwent interim PET/CT, and 21 of them (33.3%) showed a positive PET/CT outcome (scores 4–5). The univariate analysis revealed that patients with scores 1–3 on interim PET/CT were significantly associated with superior survival (PFS, *p* = 0.003; OS, *p* = 0.002). Thirty patients underwent end-of-treatment PET/CT after completing their first-line treatment. In accordance with the interim PET/CT outcome, patients with scores of 1–3 on end-of-treatment PET/CT were associated with superior survival (PFS, *p* < 0.001; OS, *p* = 0.007).

According to the univariate analysis for PFS and OS, clinical variables considered prognostic factors are summarized in Table 2. The results showed that disease stages II and III-IV, presence of B symptoms, elevated LDH, elevated β2-MG, Hb < 120 g/L, SUVmax≥9.5, interim PET/CT DS score 4/5, and end-of-treatment PET/CT DS score 4/5 were significant factors for unfavorable PFS and OS.

**Table 2:** Univariate analysis of variables predictive of PFS and OS

	PFS		OS	
Variables	HR (95% CI)	*P* value	HR (95% CI)	*P* value
Sex (Male vs. Female)	1.273 (0.831–1.948)	0.267	0.917 (0.541–1.555)	0.747
Age (≤60 vs. > 60)	1.470 (0.843–2.565)	0.174	1.296 (0.658–2.553)	0.453
Ann Arbor stage		< 0.001		< 0.001
I	Reference		Reference	
II	1.894 (1.072–3.348)	0.028	2.123 (1.027–4.390)	0.042
III-IV	3.843 (2.247–6.573)	< 0.001	4.998 (2.551–9.791)	< 0.001
Primary site (UADT vs. Extra-UADT)	1.388 (0.754–2.554)	0.292	1.612 (0.818–3.179)	0.168
B symptoms (No vs. Yes)	1.703 (1.109–2.616)	0.015	1.778 (1.055–2.995)	0.031
LDH (Normal vs. Elevated)	3.014 (1.928–4.711)	< 0.001	3.923 (2.267–6.791)	< 0.001
β2-MG (Normal vs. Elevated)	2.486 (1.639–3.772)	< 0.001	2.788 (1.687–4.609)	< 0.001
Hb (≥120 g/L vs < 120 g/L)	2.157 (1.388–3.352)	< 0.001	1.879 (1.110–3.182)	0.019
SUVmax (≤9.5 vs. > 9.5)	2.971 (1.824–4.839)	< 0.001	4.033 (2.088–7.791)	< 0.001
Interim DS (1–3 vs. 4–5)	2.738 (1.393–5.379)	0.003	3.436 (1.554–7.595)	0.002
End-of-treatment DS (1–3 vs. 4–5)	6.707 (2.234–20.14)	< 0.001	5.237 (1.412–19.43)	0.007

*Abbreviations*: *CI* confidence interval, *HR* hazard ratio, *PFS* progression-free survival, *OS* overall survival, *UADT* upper aerodigestive tract, *LDH* lactate dehydrogenase, *β2-MG* β2-microglobulin, *Hb* hemoglobin, *DS* Deauville score

Backward stepwise selection using the AIC was used to identify factors for the multivariable Cox proportional hazards regression (Table 3). Disease stage II and III-IV, elevated β2-MG, elevated LDH, Hb < 120 g/L, and SUVmax≥9.5 had the strongest association with PFS. Disease stage II and III-IV, elevated LDH, elevated β2-MG, and SUVmax≥9.5 were significantly associated with unfavorable OS.

**Table 3:** Multivariate analysis of variables predictive of PFS and OS

	PFS		OS	
Variables	HR (95% CI)	*P* value	HR (95% CI)	*P* value
Ann Arbor stage				
I	Reference		Reference	
II	1.348 (0.730–2.489)	0.340	1.448 (0.670–3.128)	0.347
III-IV	2.035 (1.098–3.771)	0.024	2.499 (1.169–5.342)	0.018
B symptoms (No vs. Yes)	1.031 (0.636–1.673)	0.901	1.011 (0.567–1.802)	0.971
LDH (Normal vs. Elevated)	1.605 (0.962–2.679)	0.070	1.971 (1.057–3.677)	0.033
β2-MG (Normal vs. Elevated)	1.744 (1.114–2.731)	0.015	1.894 (1.099–3.264)	0.022
Hb (< 120 g/L vs. ≥120 g/L)	1.533 (0.937–2.507)	0.089	1.328 (0.754–2.341)	0.326
SUVmax (≤9.5 vs. > 9.5)	1.778 (1.026–3.079)	0.040	2.192 (1.064–4.514)	0.033

*Abbreviations*: *CI* confidence interval, *HR* hazard ratio, *PFS* progression-free survival, *OS* overall survival, *LDH* lactate dehydrogenase, *β2-MG* β2-microglobulin, *Hb* hemoglobin

### Nomogram construction and internal validation

**Fig. 2:**
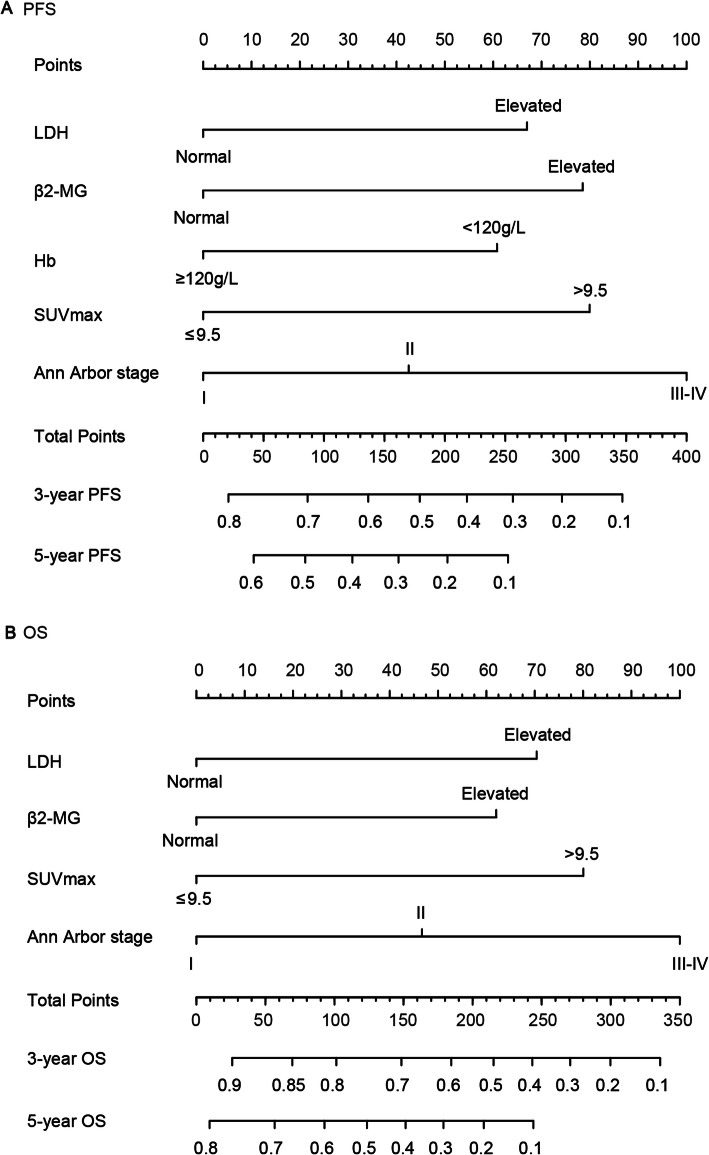
Prognostic nomograms for PFS and OS. To use the nomograms, the total points for each patient are obtained by summing the points identified on the scale for each variable. The sum of the points is plotted on the “total points” axis, and then a line is drawn downward toward the risk axis labeled “3- and 5-year PFS/OS” to determine the probabilities of the outcomes for an individual patient. PFS, progression-free survival; OS, overall survival; LDH, lactate dehydrogenase; β2-MG, β2-microglobulin; Hb, hemoglobin

The selected variables were included in the nomograms for predicting the 3-year and 5-year PFS and OS rates of patients with ENKTL (Fig. 2). Both nomograms included Ann Arbor stage (I, II, III-IV), LDH (normal or elevated), β2-MG (normal or elevated), and SUVmax (< 9.5 or ≥ 9.5). The PFS nomogram model also included Hb (< 120 g/L or ≥ 120 g/L).

**Fig. 3:**
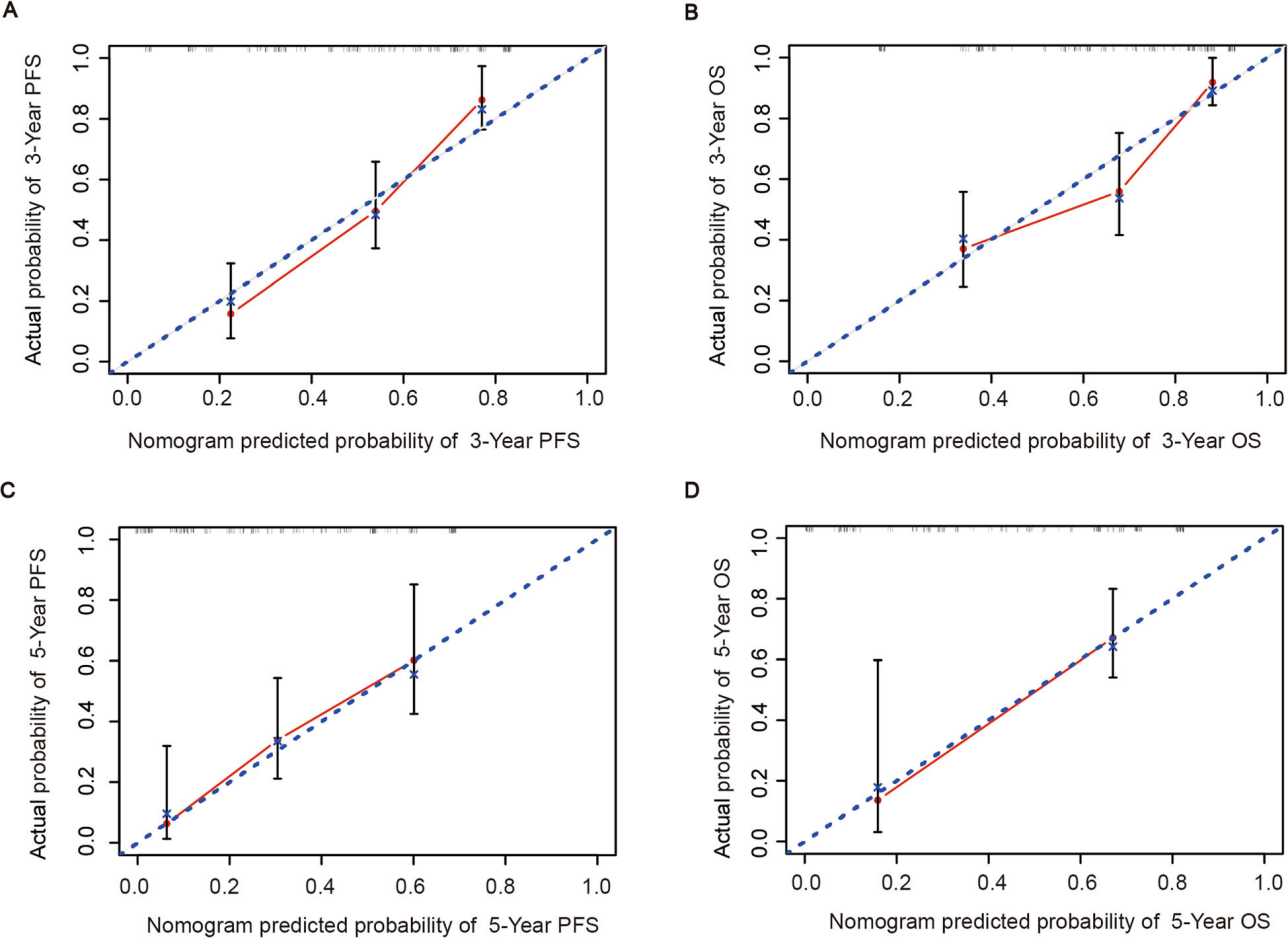
Calibration plot comparing the predicted and actual survival probabilities at 3-year PFS (**a**), 3-year OS (**b**), 5-year PFS (**c**) and 5-year OS (**d**). PFS, progression-free survival; OS, overall survival

**Fig. 4:**
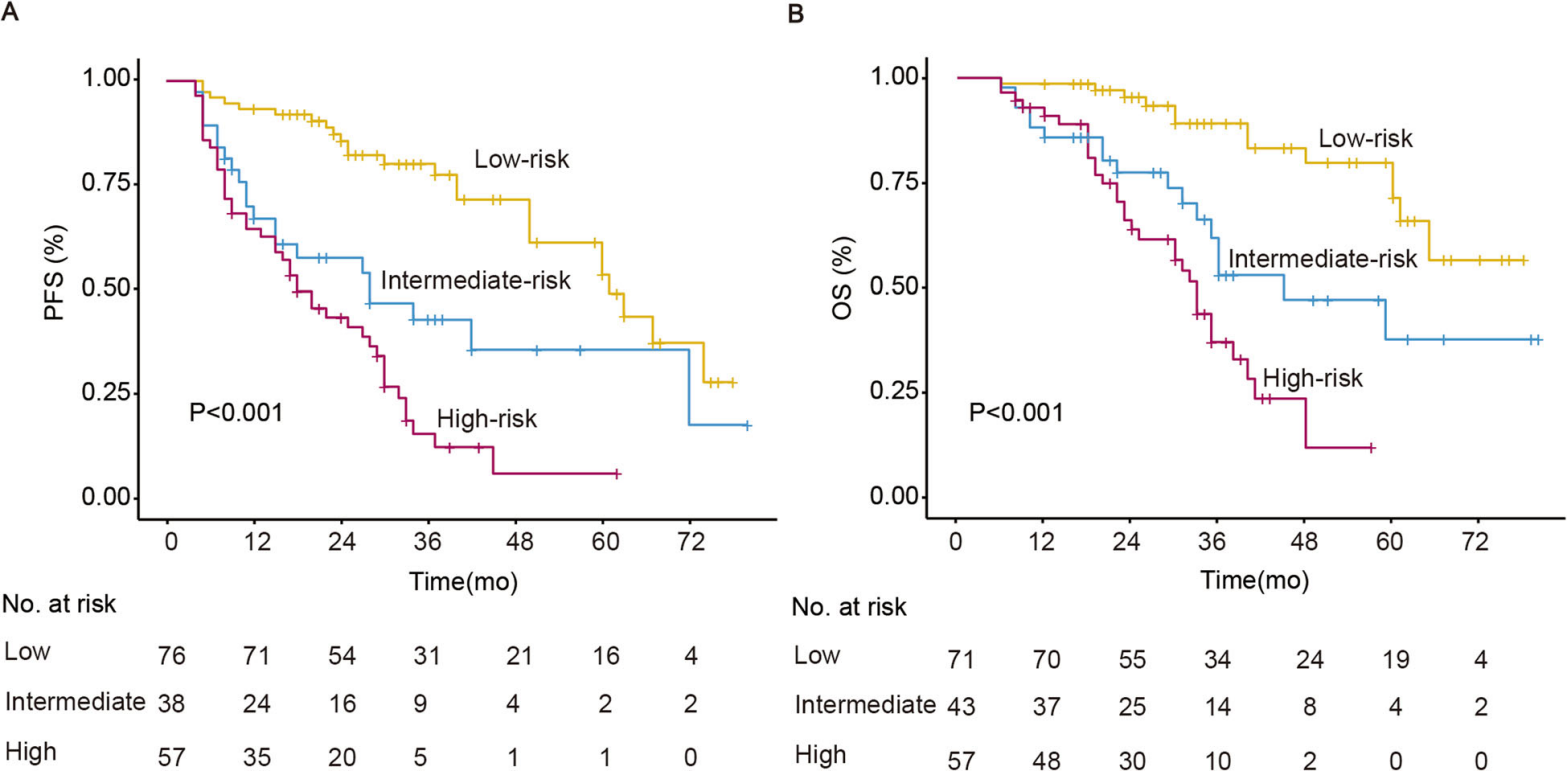
Kaplan-Meier curves demonstrating survival in patients with ENKTL according to tertiles of predicted survival. PFS, progression-free survival; OS, overall survival

For the 300 bootstrap samples, the C-index values for PFS and OS were 0.729 and 0.736, respectively. The calibration plots for 3-year and 5-year PFS/OS reported good consistency between predicted and observed probabilities for survival time (Fig. 3). To further evaluate the discriminative capacity and clinical use of the models, tertiles of total scores calculated from the nomograms were used to classify the patients into three subgroups (Fig. 4). Patients with the lowest predicted 3-year PFS (high-risk group) had worse outcomes (15.8% 3-year PFS) than patients in the low-risk and intermediate-risk groups (80.3 and 43.1% 3-year PFS, respectively) (*P* < 0.001). Similarly, patients in the high-risk group with the lowest predicted 3-year OS had substantially worse outcomes (37.0% 3-year OS) than patients in the low-risk and intermediate-risk groups (89.2 and 53.0% 3-year OS, respectively) (*P* < 0.001).

### Comparison of the predictive accuracy for PFS and OS between the nomograms and current prognostic scoring systems

**Fig. 5:**
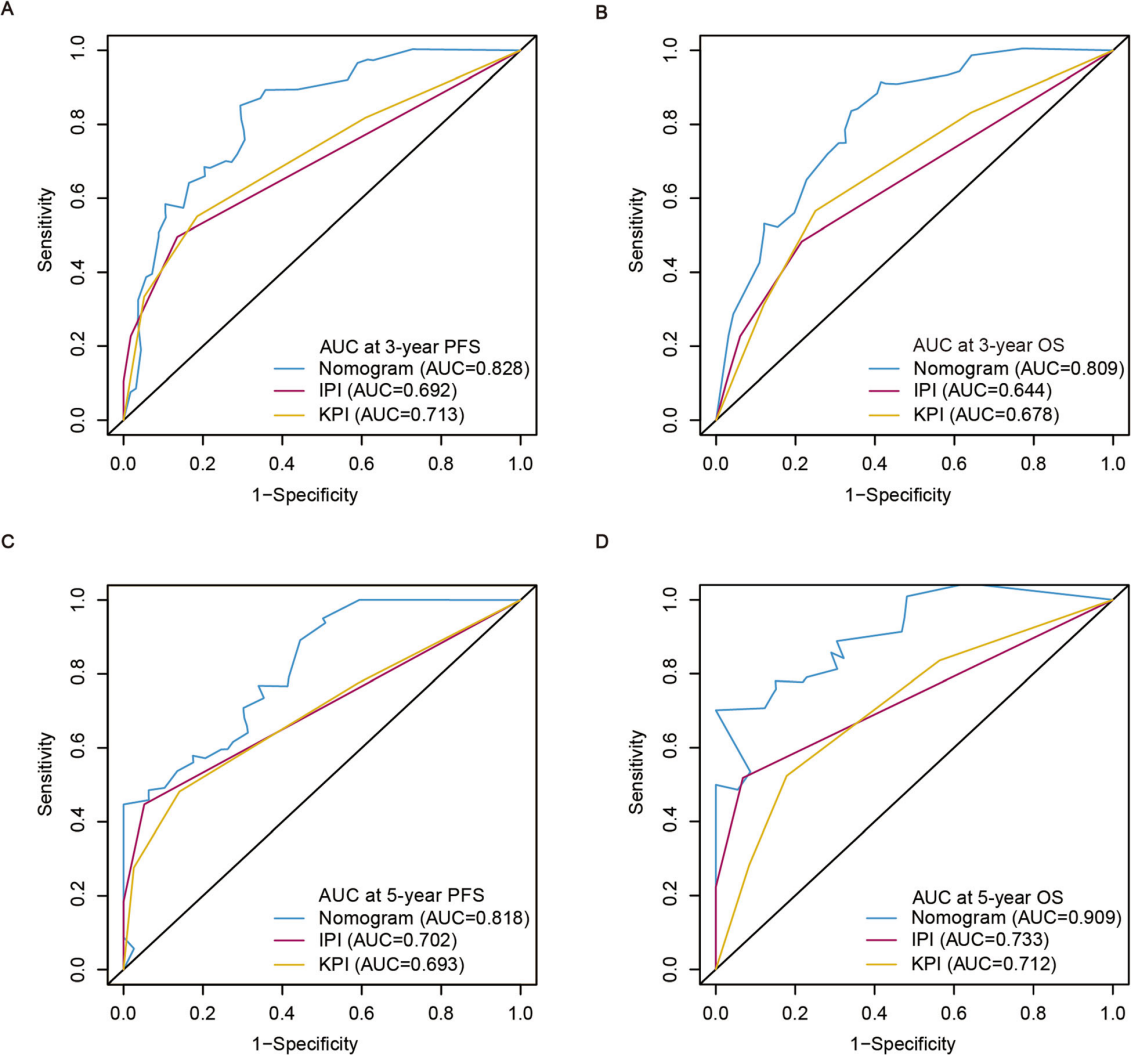
Receiver operating characteristic curves from the nomograms, IPI and KPI for 3-year PFS (**a**), 3-year OS (**b**), 5-year PFS (**c**), and 5-year OS (**d**). AUC, area under ROC curve; PFS, progression-free survival; OS, overall survival

**Fig. 6:**
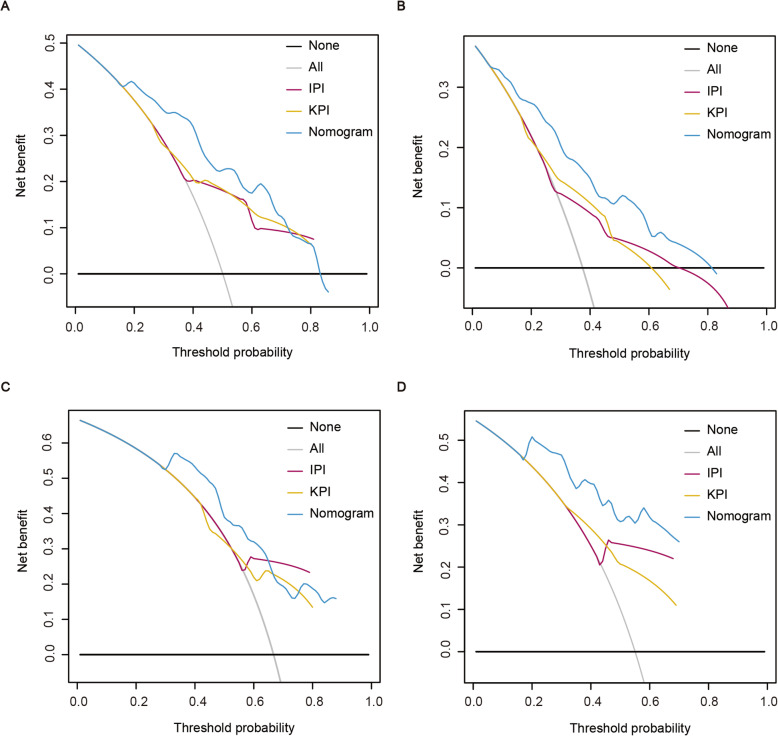
DCA from the nomograms, IPI and KPI for 3-year PFS (**a**), 3-year OS (**b**), 5-year PFS (**c**), and 5-year OS (**d**). DCA, decision curve analysis; PFS, progression-free survival; OS, overall survival

The C-index of the nomogram for PFS was 0.729 (95% CI: 0.678–0.779), whichwas significantly higher than that of the IPI (0.652; 95% CI: 0.597–0.707; *p* < 0.001) and KPI (0.644; 95% CI: 0.581–0.706; *p* < 0.001). The C-index of the nomogram for OS was 0.736 (95% CI: 0.679–0.793), which was also significantly higher than that of the IPI (0.667; 95% CI: 0.598–0.736; *p* < 0.001) and KPI (0.669; 95% CI: 0.600–0.738; *p* < 0.001). Similarly, Our results showed that the AUCs of our prognostic nomograms were higher than that of the IPI and KPI (Fig. 5). Besides, DCA showed that both the predictive accuracy of prognostic nomograms were better than the IPI and KPI (Fig. 6). These results suggested that the nomograms were more accurate and useful tools for the prognosis of PFS and OS in patients with ENKTL.

## Discussion

In this study, we established nomograms for predicting the 3-year and 5-year PFS and OS of patients with ENKTL based on the semiquantitative parameter SUVmax and clinical parameters. The nomograms showed good prediction accuracies, with the C-indexes for PFS and OS were 0.729 and 0.736, respectively. The nomograms exhibited good predictive ability for PFS and OS compared to IPI and KPI.

The rarity of ENKTL as a clinical entity means that it is a challenge to create predictive models. Therefore, it is not surprising that the predictive models for ENKTL in the literature vary greatly. The most common prognostic scoring systems include the IPI and KPI. The IPI appeared to lack discriminatory power to identify patients with more aggressive disease within the low-risk category [16–18]. The KPI was developed to address these issues and had better predictive discrimination than IPI score [7]. However, this system has also been reported to have limitations in ENKTL [19]. Nomograms are advanced prediction models that can provide a numerical probability of the survival outcome for an individual patient. In the present study, we established 2 nomograms that numerically predicted an individual’s PFS and OS for patients with newly diagnosed ENKTL. To the best of our knowledge, this study is the first to develop and internally validate nomograms using tumor metabolism and clinical parameters for the prognosis of PFS and OS in patients with ENKTL. Both nomograms included Ann Arbor stage, LDH, β2-MG, SUVmax. The PFS nomogram model also included Hb. Similar to previous studies [5], our nomograms displayed better accuracy compared to IPI and KPI. In contrast to the IPI and KPI, our prognostic nomograms included SUVmax, β2-MG and Hb as novel predictive factors.

Despite the heterogeneity of clinical characteristics and treatment modalities in our study, SUVmax showed significant efficacy in predicting the prognosis of ENKTL patients. Similar to our conclusion, some other groups also showed that ^18^F-FDG uptake before treatment could be as a predictor of treatment response and survival outcomes in patients with ENKTL [20]. Furthermore, Bai et al. [21]. reported that patients with high SUVmax were associated significantly with some adverse tumor characteristics, including local invasion (*p* = 0.030), high KPI score (*p* = 0.046), and resistance to primary treatment (*p* = 0.014). In addition, SUVmax was reported to correlated with Ki-67, which has been shown as one of the most reliable markers of tumor proliferative status [22]. The results indicated that SUVmax might reflect some inherent tumor features, and higher SUVmax might be associated with aggressive biological behavior, rapid tumor proliferation, and chemoresistance [21]. In order to accurately assess the tumor, we thought that adding SUVmax into the nomograms, along with conventional clinical parameters, could bring a better prognostic stratification for ENKTL patients. Chang et al. [13] showed that SUVmax was a significant predictor of OS (*p* = 0.017) but not PFS (*p* = 0.683) in multivariate analysis. Kim et al. [14] reported that SUVmax was associated with shorter PFS and OS (*p* = 0.020, *p* = 0.022, respectively) in univariate analysis. However, SUVmax was significant for PFS (*p* = 0.036) but not significant for OS (*p* = 0.111) in the multivariate analysis. The reason for the discrepancy may be due to 2 factors. First, the sample size of the previous studies was relatively small (171 patients in the present study vs 20–81 patients in the other studies). Second, the variety of PET/CT scanning systems and protocols may lead to different results of prognostic value.

Additionally, our study suggests that interim and end-of-treatment PET/CT analysis with DS showed clear efficacy for predicting the survival outcomes of ENKTL patients after univariate analysis, which was in accordance with the findings of many previous studies [23–25]. The parameters of interim and end-of-treatment PET/CT DS scores were not included in the nomogram because only a few patients performed interim and end-of-treatment PET/CT scans in our study. Therefore, more research is needed to clarify the association between interim/end-of-treatment PET/CT images and survival outcomes in ENKTL.

β2-MG is considered a subunit of the light chain of the class I major histocompatibility complex (MHC-I) and is present on the membrane surface of most nucleated cells [26]. Investigators conducting studies [27, 28] speculated that higher serum β2-MG levels correlated with the absence of MHC-I expression, which occurs frequently in cancer cells, and this facilitates cancer cell immune evasion and progression to metastases. Serum β2-MG is a readily accessible biomarker in blood and is overexpressed in some types of lymphomas [29, 30]. In accordance with the findings of previous studies [27], elevated serum β2-MG was associated with poor outcomes in ENKTL and included in the nomograms.

Tumor-associated anemia is common, especially in patients with hematologic malignancies. Previously, several studies explored the relationship between pretreatment Hb and the prognosis of patients. Wang et al. [31] reported that patients with Hb < 120 g/L had significantly inferior PFS and OS, but they did not include patients with stage III/IV ENKTL. Cao et al. [32] indicated that Hb level is a prognostic factor for patients with stage I-IV ENKTL, and the validated prognostic OS nomogram was useful to predict the outcomes of patients. However, opposite results were also reported. Because of the limited cases, a retrospective study indicated that Hb level has limited prognostic value [33]. In our study, univariate analysis showed that Hb was significant for survival outcomes. However, in the multivariate analysis, Hb was not significantly associated with OS (*p* = 0.326), and Hb had a tendency to be associated with PFS (*p* = 0.089). Ultimately, Hb was included in the PFS nomogram.

Despite of the nomograms showed good levels of accuracy for the prediction of PFS and OS, the present study still have some limitations. Firstly, The heterogeneity of treatment modalities may affect the treatment outcome. Secondly, our nomograms were validated using the bootstrap method in the same patients. An external cohort is needed to validate the proposed nomogram. Thirdly, our study was a retrospective study, so there may be a potential source of selection bias. Therefore, future validation of our findings in large-scale prospective studies is warranted. Finally, we only studied the semiquantitative parameter of SUVmax for predicting the prognosis of ENKTL patients, and we will explore other metabolic parameters such as MTV and TLG in the future study.

## Conclusions

We have established and internally validated nomograms involving SUVmax and clinical parameters that could predict the PFS and OS of patients with ENKTL. Notably, each factor included in our nomogram can be easily obtained. The nomograms could be effective tools for individualized prognostication in clinical medicine.

## Data Availability

All relevant raw data used and analyzed of this study are available from the corresponding author upon reasonable request.

## Abbreviations

ENKTL: Extranodal natural killer/T-cell lymphoma
PET/CT: Positron emission tomography/computed tomography
SUVmax: Maximum standardized uptake value
MTV: Metabolic tumor volume
TLG: Total lesion glycolysis
PFS: Progression-free survival
OS: Overall survival
C-index: Concordance index
ROC: Receiver operating characteristic
AUC: Area under the curve
DCA: Decision curve analysis
LDH: Lactate dehydrogenase
β2-MG: β2-microglobulin
Hb: Hemoglobin
WHO: World Health Organization
IPI: International Prognostic Index
KPI: Korean Prognostic Index
DS: Deauville score
HR: Hazard ratio
CI: Confidence intervals
UADT: Upper aerodigestive tract
